# Doppler sonography enhances rtPA-induced fibrinolysis in an *in vitro* clot model of spontaneous intracerebral hemorrhages

**DOI:** 10.1371/journal.pone.0210810

**Published:** 2019-01-17

**Authors:** Julia Masomi-Bornwasser, Philipp Winter, Axel Neulen, Sven R. Kantelhardt, Jochem König, Oliver Kempski, Florian Ringel, Naureen Keric

**Affiliations:** 1 Department of Neurosurgery, University Medical Center of the Johannes Gutenberg University, Mainz, Germany; 2 Institute of Medical Biostatistics, Epidemiology and Informatics (IMBEI), University Medical Center of the Johannes Gutenberg University, Mainz, Germany; 3 Institute of Neurosurgical Pathophysiology, University Medical Center of the Johannes Gutenberg University, Mainz, Germany; Nanjing University, CHINA

## Abstract

**Background:**

Transcranial Doppler (TCD) was shown to enhance intravascular fibrinolysis by rtPA in ischemic stroke. Studies revealed that catheter-based administration of rtPA induces lysis of intracerebral hemorrhages (ICH). However, it is unknown whether TCD would be suitable to enhance rtPA-induced fibrinolysis in patients with ICH. The aim of this study was to assess the potential of TCD to enhance rtPA-induced fibrinolysis in an *in vitro* clot system.

**Methods:**

Reproducible human blood clots of 25 ml were incubated in a water bath at 37°C during treatments. They were weighed before and after 6 different treatments: (I) control (incubation only), (II) rtPA only, (III) one Doppler probe, (IV) two Doppler probes placed vis-à-vis, (V) one probe and rtPA and (VI) two probes and rtPA. To quantify lysis of the blood clots and attenuation of the Doppler through a temporal squama acoustic peak rarefaction pressure (APRP) was measured in the field of the probes. Temperature was assessed to evaluate possible side effects.

**Results:**

Clot weight was reduced in all groups. The control group had the highest relative end weight of 70.2%±7.2% compared to all other groups (p<0,0001). Most efficient lysis was achieved using (VI) 2 probes and rtPA 36.3%±4.4% compared to (II, III, IV) (p<0.0001; p = 0.0002; p = 0.048). APRP was above lysis threshold (535.5±7.2 kPa) using 2 probes even through the temporal squama (731.6±32.5 kPa) (p = 0.0043). There was a maximal temperature elevation of 0.17±0.07°C using both probes.

**Conclusions:**

TCD significantly enhances rtPA-induced lysis of blood clots, and the effect is amplified by using multiple probes. Our results indicate that bitemporal TCD insonation of hematomas could be a new and safe approach to enhance fibrinolysis of ICH´s treated with intralesional catheter and rtPA.

## Introduction

Spontaneous intracerebral hemorrhage (ICH) occurs with an incidence of 10–30 / 100 000 per year in western countries [1,2]. Cerebral damage is caused directly by the bleeding and by second-line injury, which is induced by perihematomal edema (PHE) [3] and neuropathic products which appear during the inflammatory process [1].

Despite its significant impact, debate continues about the best treatment strategies. A clinical study demonstrated the importance of a fast reduction of intracerebral pressure [4], however, the randomized prospective STICH-trials comparing surgery with best medical treatment showed improved outcome after surgery only for a subgroup of patients with lobar hematomas close to the surface [5–7].

Recombinant tissue-type plasminogen activator (rtPA) is particularly used for the dissolution of ischemic vessel occlusions. It leads to an activation of the key enzyme of the fibrinolysis, plasminogen to plasmin. The main task of plasmin is the splitting of fibrin [8,9].

A promising minimally invasive technique for volume reduction of ICH is lysis of the intracerebral hematoma by application of recombinant tissue plasminogen activator (rtPA) [10–12] through an intralesional catheter placed stereotactically, which is also used to drain the hematoma after rtPA administration. Concerning the optimal rtPA schedule we found 1 mg with an incubation time of 15 min to be sufficient independent from ICH size in our recent *in vitro* study [13].

The MISTIE II trial (phase II RCT) investigated the effectiveness of this therapy and found a sufficient ICH volume reduction in general. In a small subgroup of patients the volume reduction correlated with a better clinical outcome [3,14]. Currently the MISTIE III trial (phase III RCT) has concluded and is being analyzed. The need to optimize this treatment method remains. In studies on ischemic stroke transcranial 2 MHz Doppler ultrasound (TCD) was found to enhance rtPA-induced fibrinolysis of endovascular thrombi [15–20]. Mechanisms like acoustic streaming are known to facilitate the streaming of rtPA through the fibrin mesh into the clot achieving a higher effectiveness of rtPA [21,22]. However, it remains unknown whether non-invasive TCD could also be used to accelerate lysis of ICH. In a clinical study on treatment of ICH, catheter-based rtPA lysis of ICH was accelerated by application of an intralesional ultrasound catheter (2 MHz) compared to application of rtPA alone [2]. In line with these findings, in an *in vitro* study with a 10 MHz ultrasound catheter, we found that combination of intralesional ultrasound and rtPA is effective especially for hematomas older than 24 hours [23], which respond worse to application of rtPA [13]. However, insertion of an intralesional ultrasound catheter would be minimal invasive. Therefore, the extern application of ultrasound using transcranial probes would be advantageous. In the future a sufficient acoustic peak rarefaction pressure (APRP), which is the most important factor for effective sonothrombolysis [21,24,25], could be achieved by application of multiple Doppler probes, placed by a navigation system to guide the hematoma. The combination of TCD with image guidance systems allows a high accurate detection [26].

In the present proof of principle study we therefore set out to investigate whether 2 MHz TCD could be used to enhance rtPA-induced hematoma lysis in an *in vitro* clot system of ICH, and whether rtPA-induced hematoma lysis is further enhanced by two TCD probes, a scenario that could easily be transferred to clinical application.

## Material and methods

### Production of blood clots for the *in vitro* clot system

Our previously well-established *in vitro* clot system, which proved to be highly reproducible in its weight, structure and density [13,23], was used for comparison of spontaneous lysis, rtPA-induced lysis, sonothrombolysis with one and two Doppler probes and combined treatment and different probe distances and lysis radius.

We collected 3 x 25 ml blood of each healthy volunteer with the help of sterile venipuncture into 20 ml syringes (BD Discardit, Germany). In total 23 healthy volunteers donated blood for 81 clots, while some of them several times. Each clot consisted of 25 ml blood, so that every donor provided 3 clots at a time: One control clot and two treatment clots. The condition was the non-consumption of aspirin or other coagulation inhibitors for at least 5 days. 10IE Thrombin (bovine plasma thrombin, Sigma, Germany; final concentration 10IE/500 μl) were added to the remaining 25 ml blood, carefully mixed and incubated for 1.5 hours at 37°C in a heat cabinet (Heraeus Instruments, Germany). Afterwards, the liquid and solid part of the blood clots were separated by a fine mesh and then weighed separately and were now ready for randomization into different treatment groups (S1 Fig).

### Experimental setting

In order to protect the blood clot from water, the solid and liquid part of the clot were put into a balloon: The opening of the balloon was cut and dilated to create a larger corridor. This allowed a gentle placement of the clot in the bottom of the balloon. Afterwards a filament was used for water tightly closure of the opening.

We produced a water bath (37°C) where the Doppler probes are attached in a depth of 10 cm and fixed in a Styrofoam board.

In experiments with one probe, the blood clots were installed at a distance of 5 cm from the probe. For all experiments probe 1 and probe 2 were tested in order to exclude differences of these two probes. By using 2 orthogonally placed Doppler probes, a distance of 14 cm between the probes was chosen, the clot was fixed at a distance of 5 cm to one probe and 9 cm distance to the other probe. The distance of 5 cm has been selected in order to reflect the removal of the typical localization of an ICH, the basal ganglia and the acoustic temporal window of the affected side. We chose the distance of 14 cm between the probes because of the following reason: We assumed an average head diameter of 14 cm: from one temporal window to the other.

After the respective treatment, the solid parts of the clots were weighed [g]. By dividing the solid clot weight at the end of the treatment from its initial weight, the relative end weight was calculated for further statistical analysis.

### Comparison of spontaneous lysis, rtPA lysis, sonothrombolysis with one and two Doppler probes and combined treatment

For this experimental series we divided 45 blood clots into 6 treatment groups (n = 6 per group). 15 probands supplied blood. In the first group, the control group blood clots (n = 15) were incubated in the balloon for one hour without treatment. Blood clots of group 2 were treated with one Doppler probe for one hour. The third group was treated with 1 mg rtPA. A concentration of 1mg rtPA/ ml, was detected as an optimal dose [13]. One mg of rtPA was added to the blood clot in the balloon after incubation. Treatment time was also one hour in the water bath (37°C). In clots of group 4, one mg of rtPA was applied and the treatment with one probe started (one hour). Group 5 was treated with both Doppler probes as described above. Group 6 received the maximal enhanced treatment with both probes and 1 mg rtPA.

### Probe distance and lysis radius

We defined autolysis when the relative weight of approximately 70% was reached as in the control group. In order to find sonothrombolysis radius, another 36 clots were treated by one Doppler probe as described above in a growing distance of 1 cm steps starting at a distance of 5 cm (target point) until a mean relative weight of approximately 70% was reached. For these experiments n = 3 clots were used for each distance.

### APRP measurement during blood clot experiments

In addition, before and after each treatment of the blood clots with the Doppler, APRP measurements were performed by piezoelectric pressure transducer (PVDF Transducer M60-3L, Dr. Müller Instruments, Germany) and an oscilloscope (DSO-1062D digital-oscilloscope, Voltcraft, Switzerland). At the point where the pressure peak was detected, the blood clots were positioned and fixed cautiously. Therefore, we were able to guarantee a perfect localization of the blood clots and the optimal alignment of the Doppler probes. Consequently, the TCD-exposure of the blood clots was always highly similar. After each treatment, another measurement was done to verify the correct alignment of the TCD and location of the clot.

### APRP measurement in order to evaluate lysis power and lysis threshold

Based on previous efforts, we were aware, that lysis is possible at a distance of 5 to 10 cm distance from the Doppler probe, because the relative weight after treatment was lower than 70%, which was defined as the lysis threshold. In order to quantify lysis, APRP was measured as described above in a distance of 5, 6, 7, 8, 9, 10 cm from probe. Every APRP measurement was performed three times by two independent investigators (21 total measurements).

In order to quantify a possible enhancement of lysis, 9 APRP measurements (n = 3 per group) were repeated at the target point (5 cm) using probe 1 or 2 or both probes. These values were related to the lysis threshold. To assess a potential difference of ultrasound effect in different tissues, the APRP measurements (n = 3 per group; total 9 measurements) were repeated in an agarose gel (Sigma Aldrich, Germany) brain model, while the experimental setting remained identical to the water bath setting [23].

As no significant differences were found between APRP values between the water bath and in the agarose brain model, all further experiments were carried out in the water bath.

The next step was the evaluation of bony attenuation of the amplitude of the pressure waves (APRP values). For this reason we use two fresh temporal bones of a roe: bone 1 and 2 to with an average thickness of 2 mm, which is comparable to human temporal bone thickness [26]. The bones were placed just in front of the Doppler probes 1 and 2. APRP measurements (n = 3 per distance; total n = 15 measurements) were done as described before in a water bath of 37°C at the target point (5 cm in front of the probe). All combinations were tried: probe1+bone1, probe1+bone2, probe2+bone1, probe1+bone2 in order to exclude significant differences of the parameters used in this setting. After proving of the similarity of each parameter a cranial bitemporal setting was built. This setting consisted of both probes placed orthogonally in the water bath. Each probe was placed behind a temporal bone and measurements were taken at the target point. All values were related to the lysis threshold. At least all APRP measurements by the PVDF Transducer M60-3L were related to a baseline, which was created by a hydrophone (Müller-Platte Needle Probe M60-1, M60-3, Dr. Müller Instruments, Germany) in a water bath (37°C). This hydrophone is known to be more exact concerning absolute values but also more fragile concerning pressures and measurement repetitions used in our experiments.

### TCD settings

The DWL Multi-Dop T digital TCD device (Compumedics Germany GmbH, Germany) was used in a pulsed-wave mode (PRF 100 Hz) at a frequency of 2 MHz, penetration depth of 5 cm and a Spatial Peak Temporal Average Intensitiy (I SPTA) of 500 mW/cm^2^. All these settings were retained in the use of 1 or 2 probes. When using both Doppler probes ultrasound was synchronically pulsed. For using the Doppler under water we used a sterile sonography probe drape. We chose an insonation time of one hour as detected in a previous study [23].

### Temperature measurement while ultrasound application

To exclude potential side effect by heating, temperature was measured in the agarose brain phantom. The two Doppler probes were placed orthogonally in a distance of 14 cm. Right in front of one probe till the middle (7 cm) in one cm steps, temperature changes were measured by a thermometer (PH Meter, WTW GmbH, Germany) every 10 minutes till one hour of insonation. Two investigators measured temperature for all in all three times (total n = 24 measurements).

### Statistical analysis

For statistical analysis we used GraphPad Prism (version 6.0). Results of blood clot experiments were reported as mean ± standard deviation (SD). Results of APRP and temperature measurements were reported as mean ± standard error (SE). Comparisons were realized by ordinary One Way Analysis of Variance. Statistical significance was defined as two-sided p-values below 0.05. For the post-hoc comparison we used the Tukey-Kramer method and adjusted p-values were reported.

### Ethical approval

This study was approved by the local Ethical Committee of Rhineland palatinate. Informed written consent was obtained from the voluntary blood donors.

## Results

### Comparison of spontaneous lysis, rtPA-induced lysis, sonothrombolysis with one and two Doppler probes and combined treatment

**Fig 1:**
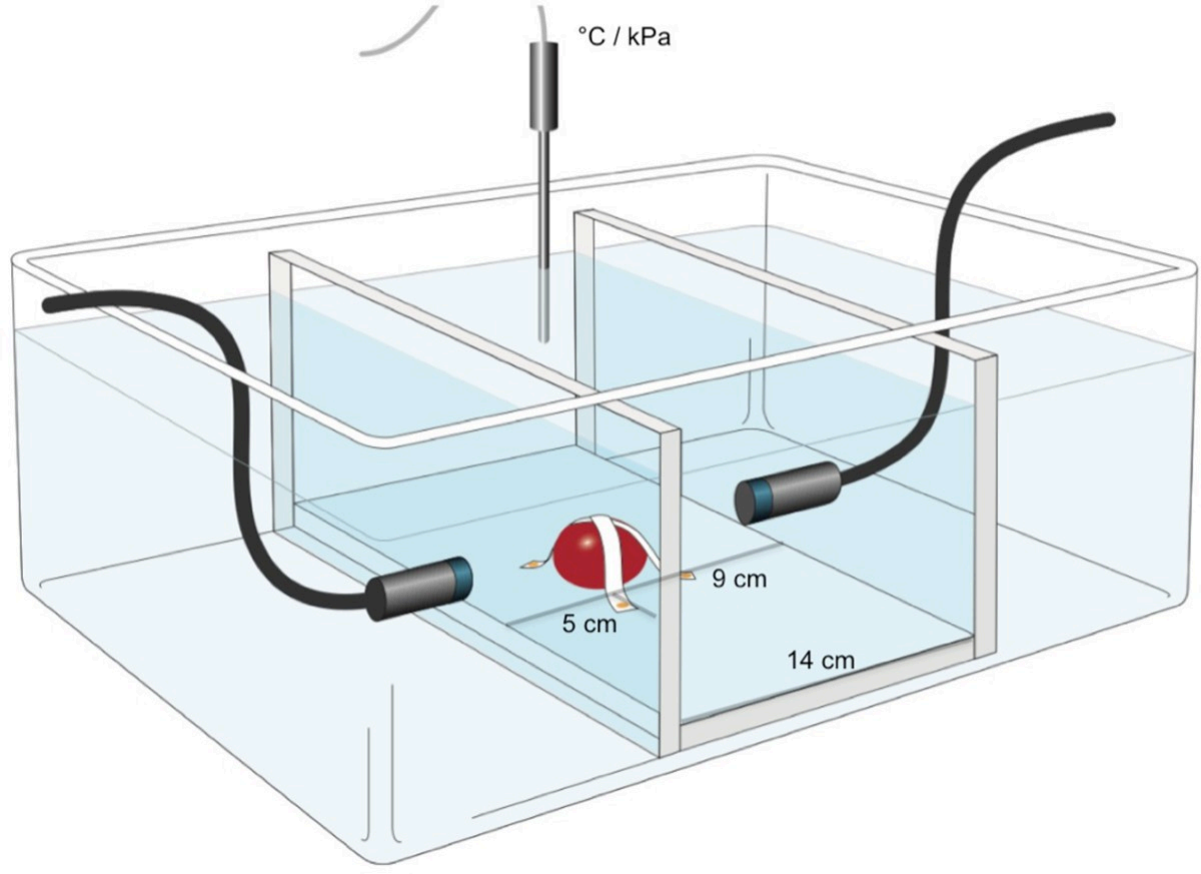
Experimental setting. All blood clots were submerged and fixed 10 cm below the water surface in a water bath (37°C), protected water tightly in a balloon in order to imitate an intracranial setting. The Doppler probes placed vis-à-vis were fixed in a Styrofoam board targeting the blood clot. Using one probe the clot was installed at a distance of 5 cm from the probe, which is a typical distance of an ICH of the basal ganglia to the temporal bone window. In case of using 2 probes, they were installed orthogonally and the second probe was fixed in a Styrofoam board at a distance of 9 cm to the clot, assuming a virtual head diameter of 14 cm. In the water bath temperature or APRP was measured at different target points.

**Fig 2:**
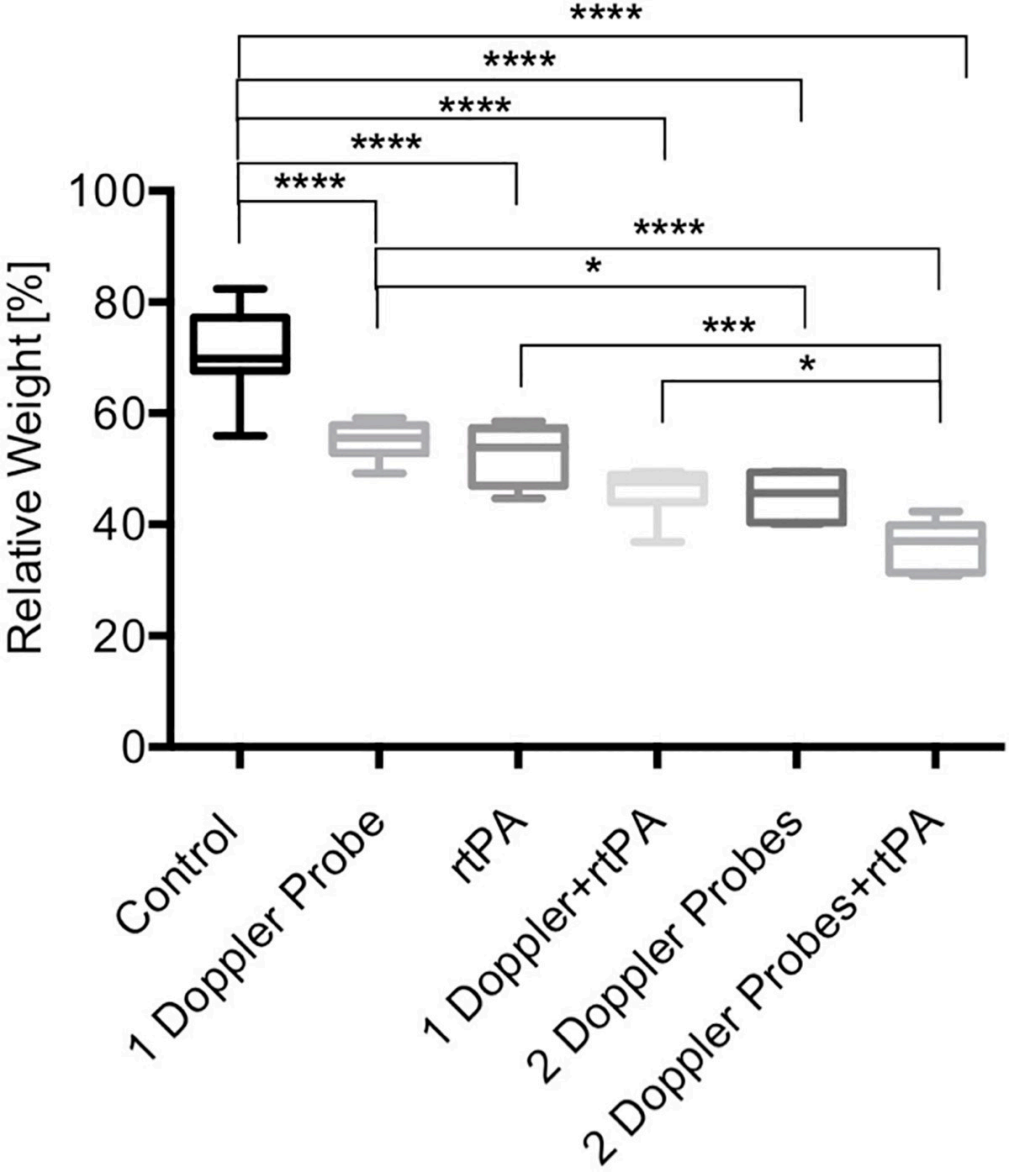
Comparison of spontaneous lysis, rtPA-induced lysis, sonothrombolysis with one and two Doppler probes and combined treatment. The box plots show the relative weight (y-axis) of the different groups (x-axis) after different treatments. ****p<0.0001, ***p = 0.0002, group 4 (1 Doppler+rtPA) versus group 6 (2 Doppler probes+rtPA) *p = 0.048, group 2 (1 Doppler probe) versus group 5 (2 Doppler probes) *p = 0.038.

Every treatment group consisted of 6 treated clots and 3 control clots (total n = 30 treated clots and 15 control clots), the mean values and standard deviation of each group are reported (see experimental setting Fig 1). The control (group 1) (incubation for 1 hour) had a relative weight of 70.2±7.2%, which was significantly higher compared to all treatment groups (p<0.0001). Group 2 (insonation with one Doppler probe) and group 3 (1 mg rtPA) had a comparable relative weight of 55.2±3.4% and 52.6±5.6% after treatment. Group 4 (rtPA+one Doppler probe) and group 5 (two Doppler probes) showed a comparable relative weight of 46.1±4.7% and 45.1±4.3%. Using two Doppler probes (group 5) was significantly more effective than one Doppler probe (group 3) (p = 0.038). The combination of two oppositely placed Doppler probes and rtPA (group 6) achieved the significantly highest lysis rate: relative weight 36.3±4.4% compared to treatment group 2 (one Doppler probe), group 3 (rtPA) and group 4 (rtPA+Doppler) (p<0.0001; p = 0.0002; p = 0.048) (Fig 2, S1 Table).

### Lysis radius

**Fig 3:**
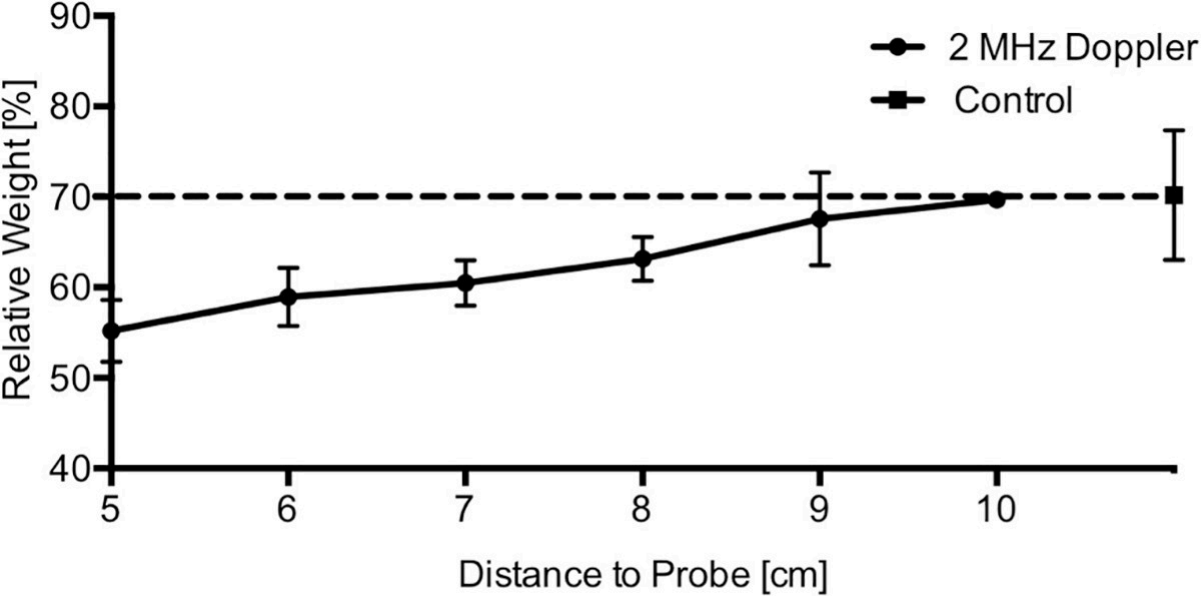
Lysis radius. The round dots symbolize the mean with standard deviation of relative clot weight (y-axis) after insonation with different distances to probe (x-axis). The square shows the mean and standard deviation of the control group 70.2±7.2%, which is defined as lysis threshold, shown as a dotted line.

Clot weight (n = 3 per distance, total n = 36) increased with growing distance to probe: 5 cm: 55.2±3.4%: 6 cm: 58.9±3.2%; 7 cm: 60.5±2.5%; 8 cm: 63.1±2.4%: 9 cm: 67.6±5.1%; 10 cm: 69.7±0.5% (mean and SD). Spontaneous lysis was defined as relative clot weight after one hour of incubation without any treatment: 70.2±7.2% (control group). Hence lysis radius was 10 cm (Fig 3, S2 Table).

### APRP values with increasing distance to probe

**Fig 4:**
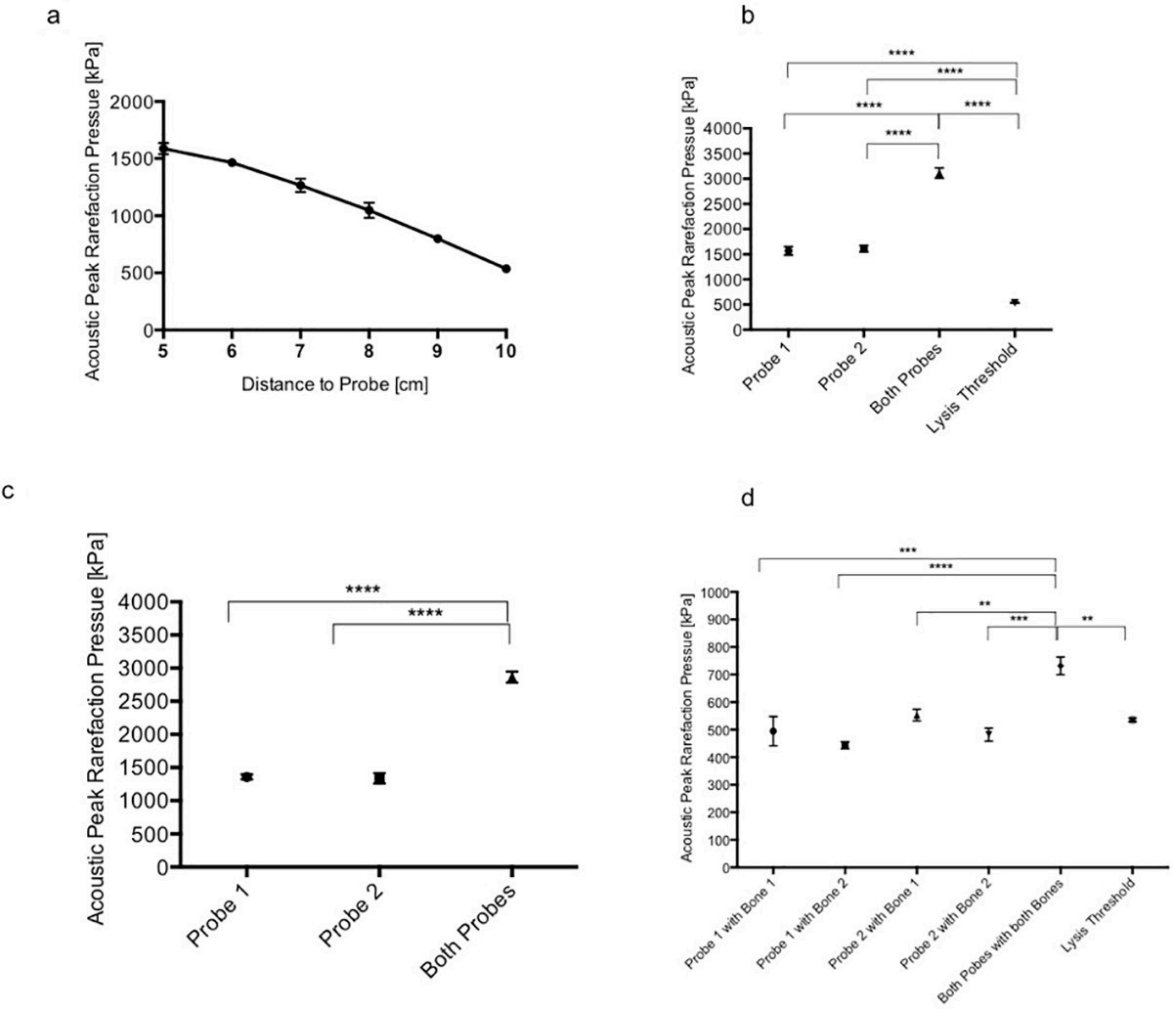
Acoustic Peak Rarefaction Pressure (APRP) measurement. **(a)** APRP values (y-axis) of 2 MHz Doppler are measured in different distances to probe (x-axis) (mean, SE) (n = 3). At the outer edge of lysis radius (10 cm) 535.5±7.2 kPa was measured (lysis threshold). **(b)** Acoustic Peak Rarefaction Pressure (APRP) measurement (n = 3) using one or two Doppler Probes in a water bath. Means and SE of APRP measurements are shown using Doppler probe 1 or 2 or both of them. These measurements were related to lysis threshold, **** p<0.0001. **(c)** Acoustic Peak Rarefaction Pressure (APRP) measurement (n = 3) using one or two Doppler Probes in an agar brain model. Means and SE of APRP measurements are shown using Doppler probe 1 or 2 or both of them,**** p<0.0001. These values were similar to the measurements in the water bath. **(d)** Acoustic Peak Rarefaction Pressure (APRP) measurement (n = 3) using one or two Doppler probes considering bone attenuation. Means and SE of APRP measurements are shown using ultrasound probe 1 and probe 2 attenuated by a temporal bone 1 and 2. All combinations were performed and compared to the use of both probes and bones placed vis-à-vis (cranial setting) and to lysis threshold. ****p<0.0001, probe1+bone1 versus both probes+both bones: ***p = 0.0007, probe2+bone2 versus both probes+both bones:***p = 0.0004, probe2+bone1 versus both probes+both bones: **p = 0.0091, both probes+both bones versus lysis threshold:**p = 0.0043.

APRP values (n = 21 measurements) decreased almost linearly with increasing distance to probe. In a distance of 5 cm 1587.8±48.2 kPa (mean and standard error) was measured (n = 6). Measuring in a 6 cm distance (n = 3) a value of 1466.4±18.9 kPa was shown; 7 cm (n = 3): 1266.1±57.8 kPa; 8 cm (n = 3): 1048.2±68.1 kPa; 9 cm (n = 3): 799.1±27.5 kPa. At the outer edge of lysis radius (10 cm) (n = 3) 535.5±7.2 kPa was measured (Fig 4A).

### APRP measurement using one or two Doppler probes

In a water bath APRP measurement (n = 3 per distance, total n = 18) at the target point of 5 cm from Doppler probe 1 was 1567± 83.5 kPa (mean, SE). This was similar to probe 2: 1609±65.0 kPa. Using both probes vis-à-vis at the target point: 5 cm from probe 1 and 9 cm from probe 2, APRP was doubled and therefore significantly higher than using one probe: 3223±100.1 kPa, p>0.0001. All measurements were significantly above lysis threshold of 535.5±7.2 kPa measured at the outer edge of lysis radius (10 cm) before, p>0.0001 (Fig 4B). The same measurements were repeated in an agar brain model, which resulted in similar findings: Probe 1: 1360±37.4 kPa; Probe 2: 1339± 78.4 kPa; both probes: 2864± 82.4 kPa (Fig 4C). APRP values were only slightly higher in water than in the agarose brain model, but this was not significant.

### APRP measurement using one or two Doppler probes considering bone attenuation

In a water bath APRP was measured (n = 3 per distance, total n = 15) at the target point (5 cm distance to probe) but this time insonation was performed through a temporal bone (bone 1, bone 2). Attenuation was approximately 70%: group 1 (probe1+bone1) showed an APRP of 495±53.1 (mean, SE). Similar values were measured using every combination of Probe 1,2 and bone 1,2: group 2 (probe 1+bone2): 443.7±12.7; group 3 (probe 2+bone1): 552.6±21.4 kPa; group 4 (probe2+bone2): 482.1±23.5 kPa. Rebuilding a cranial setting (Fig 1) APRP measured at the target point using both probes vis-á-vis through both temporal bones also placed vis-à-vis in a distance of 14 cm was 731.6±32.5 kPa, which is significantly above lysis threshold of 535.5±7.2 kPa, p = 0.0043. This setting showed a lowering of ultrasound amplitude (APRP) from approximately 80% by temporal bones. APRP of the cranial setting was significantly above APRP using one probe and one bone: group 1: p = 0.0007; group 2: p>0.0001; group 3: p = 0.0091; group 4: p = 0.0004 (Fig 4D).

### Temperature measurement during insonation

Using both Doppler probes (Fig 1) in a distance of 0 to 7 cm from 1 ultrasound probe during one hour a maximal temperature elevation of 0.17±0.07°C (mean, standard error) was found in a distance of 5 cm from probe (Table 1).

**Table 1:** Temperature measurement during insonation (n = 3 per distance, total n = 24).

Distance to probe [cm]	Mean [°c]	Standard error [°c]
0	0.03	0.34
1	0.13	0.12
2	0.1	0.06
3	0.13	0.07
4	0.13	0.03
5	0.17	0.07
6	0.1	0.1
7	0.1	0.1

## Discussion

Several studies have reported the efficacy of catheter-based treatment of ICH with rtPA [11,12,27–29]. One clinical trial showed an enhancement of rtPA-induced hematoma lysis by additional use of an intralesional 2 MHz ultrasound catheter [2]. Here we demonstrate, to the best of our knowledge for the first time, a significant sonothrombolysis with transcranial 2 MHz Doppler probes commonly used for diagnostic transcranial Doppler sonography in an *in vitro* clot system of ICH. In clinical use, the greatest advantages compared to intralesional ultrasound catheters would be the non-invasive nature of transcranial ultrasound and lower costs. The typical localization of the intracerebral hemorrhage are the basal ganglia [30]. These can be reached easily by TCD through the acoustic temporal window [31].

We could show that 2 MHz, single-element pulsed-wave ultrasound leads to an increase of thrombolysis. As expected, the combination of rtPA and ultrasound had a greater lytic efficacy than rtPA alone, which is in line with several clinical studies concerning sonothrombolysis of ischemic strokes [15–19]. It is known that ultrasound alone even disaggregates fibrin fibers temporarily [32]. Ultrasound not only supports the streaming of rtPA into the clot but also enlarges the supply of its binding partner plasminogen [21,22]. In our previous study we could morphologically show the irreversible rarefication of the fine fibrin mesh using the combination of rtPA and ultrasound [23]. Several studies suggest APRP for being the main factor enhancing ultrasound thrombolysis [21,24,25]. These data correlate rather well with this experimental study. Orthogonally placed Doppler probes increased APRP and enhanced lysis significantly compared to the use of only one probe. The best lysis result was achieved using 2 probes in combination with rtPA (Fig 2).

We found a lysis radius for 2 MHz Doppler of 10 cm, a distance that would be sufficient for cranial use. APRP measurements were also performed to quantify the lysis potential. APRP measurements at the outer edge of lysis radius (10 cm) revealed a lysis threshold of approximately 500 kPa (Fig 4A). This value is comparable to the lysis threshold measured previously by us *in vitro*, where ultrasound frequencies of 5.5 up to 10 MHz where tested [23]. With growing distance to the Doppler probe APRP decreased almost linearly (Fig 4A). Measurements in the water bath and in the agar brain phantom were comparable (Fig 4B and 4C). We could show that ultrasound amplitude using one probe (approximately 1500 kPa) can be doubled, when two probes are used orthogonally targeted at one point (approximately 3000 kPa) (Fig 4B and 4C), which explains the significant enhancement of hematoma lysis using 2 probes.

This experimental setting does not include attenuation of ultrasound by the temporal bone. It is important to note that clinical studies on ischemic stroke showed a significant lysis enhancement through 1 transcranial 2 MHz Doppler probe applied through the temporal bone [15–19]. Our experiments with simulation of transcranial insonation with a bone sample show that using one probe through a temporal bone only reached lysis threshold marginally (Fig 4D), bone-attenuation of ultrasound was approximately 70% to 80%, which is comparable to observations in other studies [15,33]. Using two orthogonally placed probes through two bones, which would be comparable to bitemporal use of TCD probes lead to an increase of the ultrasound amplitude (APRP) by approximately 30%, which was significantly above lysis threshold.

A study of Bardon and coworkers [34] in patients with ischemic stroke showed that using two probes and bilateral monitoring is safe but not more effective than standard sonothrombolysis in acute ischemic stroke patients with MCA occlusion.

The correct position of the hand-held probe in this study was confirmed by using the detection of the preocclusive flow in the MCA and a typical “click”in Doppler mode [34]. Perhaps a reason could be the inaccuracy and operator dependency [35] of a hand-held tool targeting a sample volume, which was set at 10 mm [34]. Navigated Doppler probes [26] could overcome the problem of targeting the hematoma using an image guidance system. These image guidance systems are a common tool used for intraoperative neuronavigation [26]. A study from Neulen et al. approved this in the detection of vasospasms by image-guided TCD, which was higher compared to conventional TCD with a high accuracy (deviation<3mm) [26]. Further advantages of image-guided control are an improvement of intra- and inter-investigator variation especially for inexperienced investigators and in combination with poor bone windows [26]. Clinical studies in patients with ICH will be needed to examine whether 1, 2 or more Doppler probes produce optimal sonothrombolytic effects.

We explain improvement of lysis with the combination of ultrasound and rtPA with heterogeneous effects, presumably based on reversible alteration of the fibrin structure [32], which improves the drug transport and results in increased binding of rtPA inside the blood clot by acoustic streaming [21,22]. This results in a rarefaction of the fine fibrin mesh inside the clot [23]. This ultrasound induced mechanism also increases the enzymatic fibrinolysis by transport of the rtPA binding partner plasminogen from the outer plasma into the clot and also in transverse direction [21].

Side effects of cranial ultrasound use are an important theme. Loss of blood-brain-barrier, brain edema, necrosis are known as possible risks caused by ultrasound [36,37]. In a clinical study transcranial low-frequency ultrasound (300 kHz) was applied in order to enhance rtPA lysis of middle cerebral artery occlusion. This study was stopped because symptomatic intracranial hemorrhage occurred in several cases [18].

In our study, pulsed-wave (PW) Doppler was used. In PW Doppler mode unplanned interferences of ultrasound waves are unusual and can be excluded to cause side effects [34]. Recent studies have verified the safety of using 1 or 2 TCD’s [15–19][34]. The CLOTBUST Hands Free pilot safety study confirms the safe use of 2 hours sonothrombolysis by a head frame, which contains 18 ultrasound transducers (2 MHz, pulsed-wave, bitemporal and suboccipital) [35] in combination with rtPA in a phase III efficacy trial [38]. In our study the emitted Spatial Peak Temporal Average Intensity (I SPTA) of 500 mW/cm^2^ (during one hour) is under the threshold that is allowed by the FDA (<720mW/cm^2^) [35,38]. No relevant temperature increase could be found applying 2 Doppler probes simultaneously, which confirms the safety of this therapy (Table 1).

**Fig 5:**
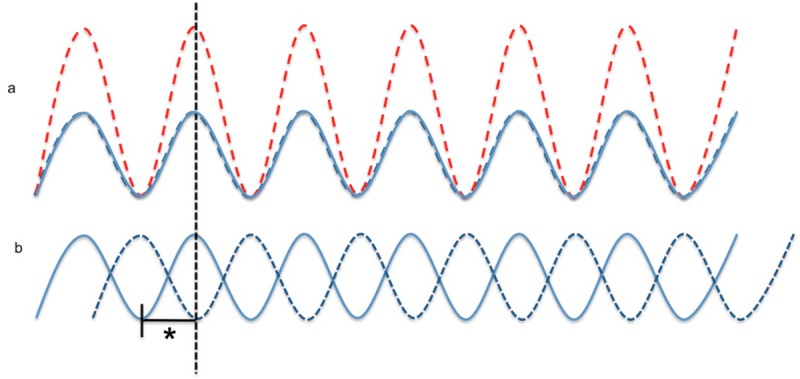
Superposition and cancellation of the ultrasound waves. **(a)** The superposition (red dotted wave) of two ultrasound waves (blue) in phase is illustrated. The two blue waves are congruent and lead to a doubled amplitude (red dotted wave). **(b)** The two blue waves are anti-phase, which result in a cancellation of both waves. The * marks the half of the wavelength, which presents the distance between superposition and cancellation.

In order to achieve a superposition of the amplitude of the ultrasound waves by two probes with the goal to enhance lysis, it requires a precise attachment of the probes so that the ultrasound waves meat each other. If two ultrasound beams are in phase, the wave amplitude is doubled [39]. If the waves are anti-phase they cancel each other out [39]. Every case in between leads to an addition of the amplitude, which is smaller than the doubled amplitude, called “constructive interference” [39] (Fig 5A and 5B). Being aware of the complex *in vivo* situation, where Doppler probes cannot be positioned strictly orthogonally and the ultrasound waves may not be exclusively in phase leading to a doubled amplitude due to geometrical reasons, therefore the aim should be a constructive interference.

In general these results have to be interpreted with caution. We used common brain tissue models like water, agarose gel [40] and bony attenuation but we are aware of the fact that *in vivo* parameters, which influence the ultrasound waves, are more complex.

It is evident that the surrounding tissues of an ICH can influence lysis and the clot formation but this *in vitro* model can reflect the inner part of a large bleeding, which can help to optimize lysis in the future. In clinical application, the use of 2 or more Doppler probes could induce effects of addition and extinction. However, potentially unwanted effects could be controlled by appropriate technical and software modifications.

## Conclusion

In this study we could demonstrate the effective lysis of blood clots by Doppler ultrasound, which can be enhanced by using two Doppler probes realized by our reproducible *in vitro* ICH clot system. Best results were achieved using two Doppler probes and rtPA. Attenuated through the temporal bone Doppler ultrasound is just marginally above lysis threshold. The bilateral Doppler alignment leads to a safe enhancement above lysis threshold.

Consequently, the enhancement of catheter based rtPA thrombolysis of ICH by ultrasound could be applied safely and noninvasive at the bedside by using TCD. The bitemporal insonation could be a promising therapy presupposed an accurate alignment of the probes is given by an image guidance system.

## Supporting information

S1 Fig Experimental workflow. This diagram summarizes the different experimental settings.
(PPTX)

S1 Table Absolute weights after spontaneous lysis, rtPA-induced lysis, sonothrombolysis with one and two Doppler probes and combined treatment. (DOCX)

S2 Table Lysis radius, absolute weights. (DOCX)
