# Supplementary material for: Doppler sonography enhances rtPA-induced fibrinolysis in an in vitro clot model of spontaneous intracerebral hemorrhages
Source: PLoS One. 2019 Jan 17;14(1):e0210810. doi: 10.1371/journal.pone.0210810 (PMC6336417; doi:10.1371/journal.pone.0210810)
Supplement: S1 Fig — This diagram summarizes the different experimental settings. (PPTX) [file pone.0210810.s001.pptx]

## Slide 1
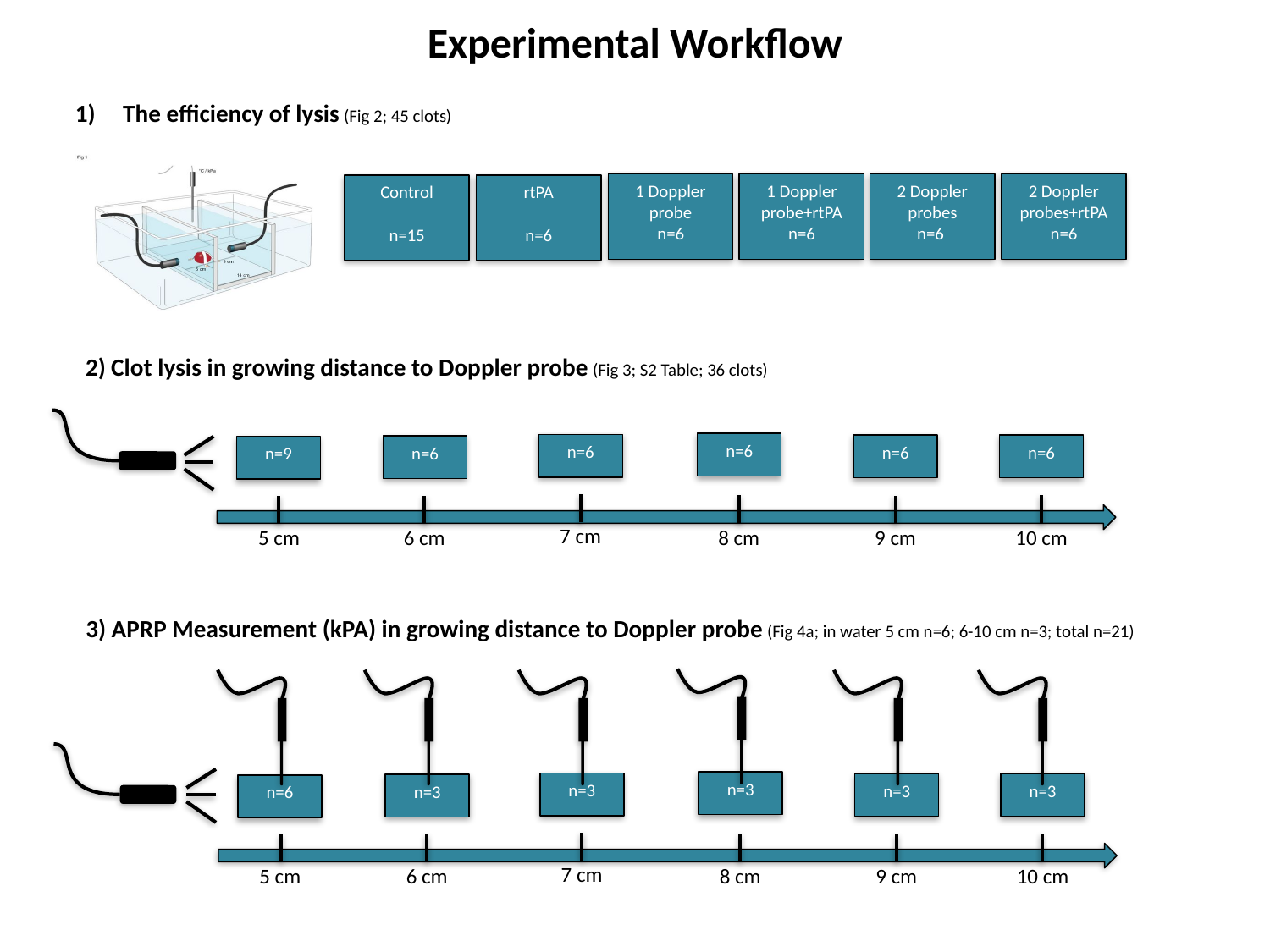

# Experimental Workflow
The efficiency of lysis (Fig 2; 45 clots)
1 Doppler probe
n=6
1 Doppler probe+rtPA
n=6
2 Doppler probes
n=6
2 Doppler probes+rtPA
n=6
Control
n=15
rtPA
n=6
2) Clot lysis in growing distance to Doppler probe (Fig 3; S2 Table; 36 clots)
n=6
n=6
n=6
n=6
n=6
n=9
7 cm
10 cm
5 cm
8 cm
9 cm
6 cm
3) APRP Measurement (kPA) in growing distance to Doppler probe (Fig 4a; in water 5 cm n=6; 6-10 cm n=3; total n=21)
n=3
n=3
n=3
n=3
n=3
n=6
7 cm
10 cm
5 cm
8 cm
9 cm
6 cm

## Slide 2
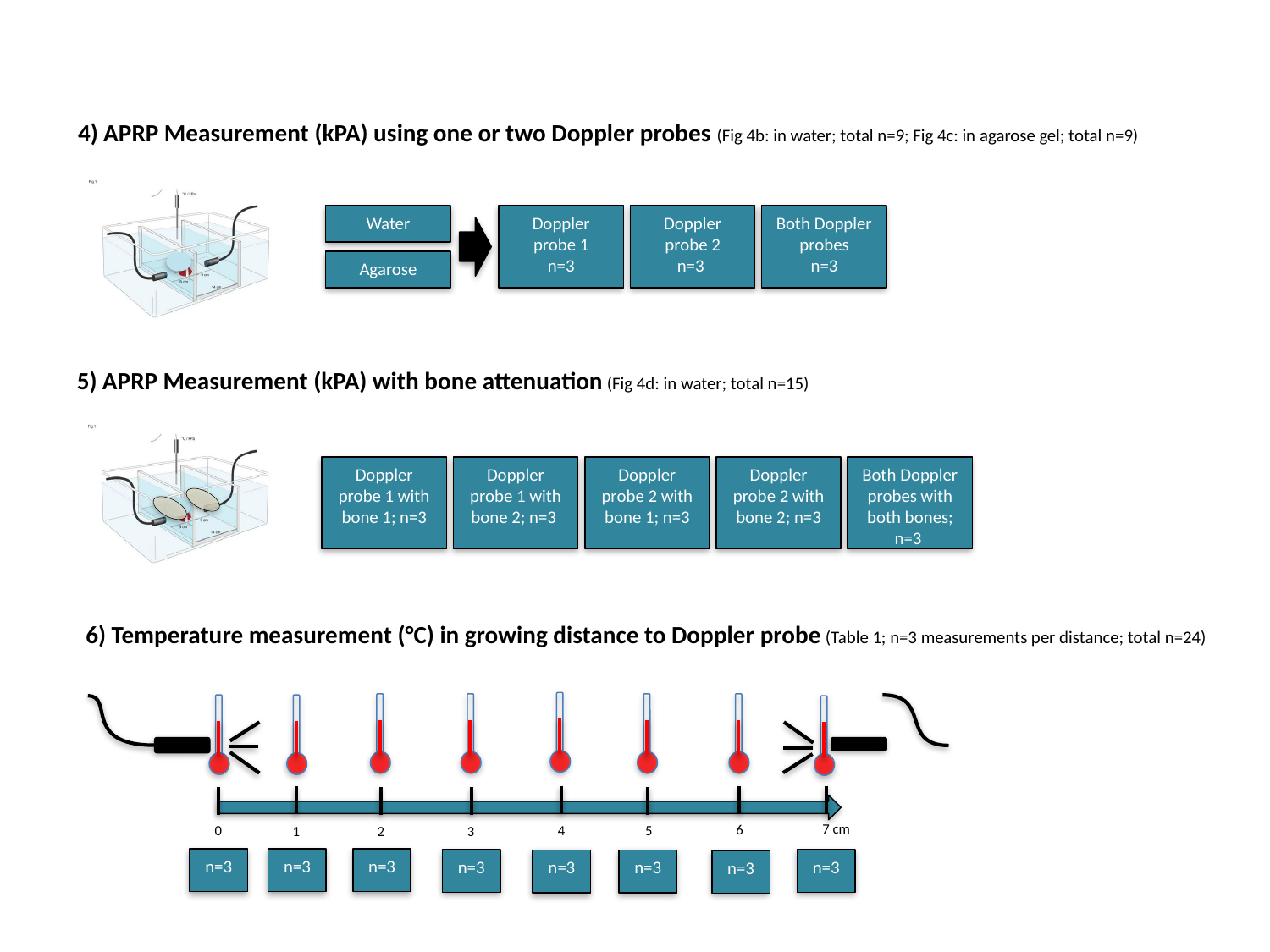

4) APRP Measurement (kPA) using one or two Doppler probes (Fig 4b: in water; total n=9; Fig 4c: in agarose gel; total n=9)
Water
Doppler probe 1
n=3
Doppler probe 2
n=3
Both Doppler probes
n=3
Agarose
5) APRP Measurement (kPA) with bone attenuation (Fig 4d: in water; total n=15)
Doppler probe 1 with bone 1; n=3
Doppler probe 1 with bone 2; n=3
Doppler probe 2 with bone 1; n=3
Doppler probe 2 with bone 2; n=3
Both Doppler probes with both bones; n=3
6) Temperature measurement (°C) in growing distance to Doppler probe (Table 1; n=3 measurements per distance; total n=24)
7 cm
6
4
0
5
3
2
1
n=3
n=3
n=3
n=3
n=3
n=3
n=3
n=3
