# Supplementary material for: Doppler sonography enhances rtPA-induced fibrinolysis in an in vitro clot model of spontaneous intracerebral hemorrhages
Source: PLoS One. 2019 Jan 17;14(1):e0210810. doi: 10.1371/journal.pone.0210810 (PMC6336417; doi:10.1371/journal.pone.0210810)
Supplement: S1 Table — (DOCX) [file pone.0210810.s002.docx]

| Control  (n=15) | 1 Doppler probe  (n=6) | rtPA  (n=6) | 1 Doppler probe + rtPA  (n=6) | 2 Doppler probes (n=6) | 2 Doppler probes + rtPA  (n=6) |
| --- | --- | --- | --- | --- | --- |
| 12.8±1.89g | 10.43±1 g | 11±1.67 g | 8.6±1.34 g | 8.13±1g | 6.6±1.8g |

**Table S1. Absolute weights after spontaneous lysis, rtPA-induced lysis, sonothrombolysis with one and two Doppler probes and combined treatment.**

(mean ± standard deviation).
