# Supplementary material for: Doppler sonography enhances rtPA-induced fibrinolysis in an in vitro clot model of spontaneous intracerebral hemorrhages
Source: PLoS One. 2019 Jan 17;14(1):e0210810. doi: 10.1371/journal.pone.0210810 (PMC6336417; doi:10.1371/journal.pone.0210810)
Supplement: S2 Table — (DOCX) [file pone.0210810.s003.docx]

**Table S2. Lysis Radius, absolute Weights.**

| Control  (n=15) | 5 cm distance to probe (n=6) | 6 cm distance to probe (n=3) | 7 cm distance to probe (n=3) | 8 cm distance to probe (n=3) | 9 cm distance to probe (n=3) | 10 cm distance to probe (n=3) |
| --- | --- | --- | --- | --- | --- | --- |
| 12.8±1.89g | 10.43±1 g | 11.93±1g | 12.23±1.3g | 11.9±1.4g | 11.77±0.86 | 12.57±2.17g |

(mean ± standard deviation).
